# Emerging Perspective: Role of Increased ROS and Redox Imbalance in Skin Carcinogenesis

**DOI:** 10.1155/2019/8127362

**Published:** 2019-09-16

**Authors:** Dehai Xian, Rui Lai, Jing Song, Xia Xiong, Jianqiao Zhong

**Affiliations:** ^1^Department of Anatomy, Southwest Medical University, Luzhou 646000, China; ^2^Department of Dermatology, The Affiliated Hospital of Southwest Medical University, Luzhou 646000, China

## Abstract

Strategies to battle malignant tumors have always been a dynamic research endeavour. Although various vehicles (e.g., chemotherapeutic therapy, radiotherapy, surgical resection, etc.) are used for skin cancer management, they mostly remain unsatisfactory due to the complex mechanism of carcinogenesis. Increasing evidence indicates that redox imbalance and aberrant reactive oxygen species (ROS) are closely implicated in the oncogenesis of skin cancer. When ROS production goes beyond their clearance, excessive or accumulated ROS could disrupt redox balance, induce oxidative stress, and activate the altered ROS signals. These would damage cellular DNA, proteins, and lipids, further leading to gene mutation, cell hyperproliferation, and fatal lesions in cells that contribute to carcinogenesis in the skin. It has been known that ROS-mediated skin carcinogenesis involves multiple ways, including modulating related signaling pathways, changing cell metabolism, and causing the instability of the genome and epigenome. Nevertheless, the exact role of ROS in skin cancer has not been thoroughly elucidated. In spite of ROS inducing skin carcinogenesis, toxic-dose ROS could trigger cell death/apoptosis and, therefore, may be an efficient therapeutic tool to battle skin cancer. Considering the dual role of ROS in the carcinogenesis and treatment of skin cancer, it would be essential to clarify the relationship between ROS and skin cancer. Thus, in this review, we get the related data together to seek the connection between ROS and skin carcinogenesis. Besides, strategies basing on ROS to fight skin cancer are discussed.

## 1. Introduction

Skin cancer is the most common type of cancer, and its incidence has gradually increased in recent years [1]. It is characterized by aberrant cell growth with a potential to invade or spread elsewhere in the body, which involves the complex process of carcinogenesis [2]. At present, the main types of skin cancer are melanoma and nonmelanoma skin cancer (NMSC), while the latter includes basal cell carcinoma (BCC) and squamous cell carcinoma (SCC). Ultraviolet (UV) exposure is one of the main factors inducing skin cancer, and cutaneous cells may be damaged directly by UV radiation or indirectly by UV-mediated reactive oxygen species (ROS) overproduction [3]. Long-term UV radiation could cause photochemical reactions or/and oxidative DNA damage, induce DNA mutation and misexpression, and trigger skin carcinogenesis [4]. UV irradiation induces the skin to produce substantial ROS, which results in nuclear DNA damage via forming a large amount of cyclobutane pyrimidine dimers (CPDs), pyrimidine (6-4), pyrimidone photoproducts, and 8-oxodG [5]. 8-oxodG, a biomarker of oxidative damage to DNA, could be removed from the damaged DNA by the enzyme human 8-oxoguanine-DNA glycosylase 1 (hOGG1). In the study, it was shown that UVB-induced ROS triggered 8-oxoguanine (8-oxoG) production and hOGG1 reduction in the skin, further damaging the DNA repair pathway, and eventually initiating cutaneous carcinogenesis [6, 7].

ROS belong to oxygen-derived small molecules including oxygen-centered radical species (e.g., superoxide (O_2_^•-^), hydroxyl (^•^OH), peroxyl (R-O_2_^•^), and alkoxyl (RO^•^)) and nonradical compounds that are either oxidizing agents or easily converted into radicals, such as hypochlorous acid (HOCl), ozone (O_3_), singlet oxygen (^1^O_2_), and hydrogen peroxide (H_2_O_2_) [8]. ROS are one of the normal products of physiological metabolism and are mainly derived from endogenous and exogenous sources [9]. Endogenous sources are primarily produced by complex I and complex III in the oxidative respiratory electron transport chain (ETC) of mitochondria [10]. Some also originate from enzymes, including NADPH-oxidases (NOXs), lipoxygenases, xanthine oxidases, nitric oxide synthases, and cytochrome p450 enzymes [11]. Apart from the previously mentioned sources, environmental stress (e.g., chemical substances, drugs, UV radiation, ionizing radiation (IR), and hypoxia) could induce ROS production. Under physiological conditions, ROS production and scavenging are in a dynamic equilibrium and the body is in a redox homeostasis at the presence of the antioxidant defense system, which is vital to normal physiological response [12]. The antioxidant defense system mainly includes an enzymatic antioxidant system (e.g., superoxide dismutase (SOD), glutathione peroxidase (GSH-Px), catalase (CAT), thioredoxin (TRX), and peroxiredoxin) and a nonenzymatic one (e.g., tripeptide glutathione (GSH), vitamins (vitamins C and E), *β*-carotene, and uric acid) [13]. Through these two antioxidant systems, oxygen radicals and nonradicals (O_2_^•-^, ^•^OH, H_2_O_2_, etc.) could be converted into H_2_O and eventually into O_2_ [14, 15].

**Figure 1:**
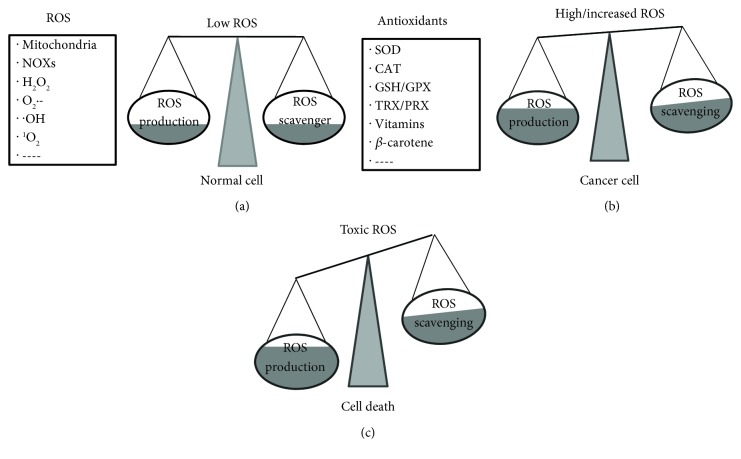
The effects of different concentrations of ROS on cells. (a) ROS, derived from mitochondria, NOXs, etc., mainly contain H_2_O_2_, O_2_^•-^, ^•^OH, and others. Scavenging involves the antioxidation system, such as SOD, CAT, TRX/PRX, and vitamins. Low doses of ROS production and scavenging are in a dynamic equilibrium, which is beneficial to the physiological function of normal cells. (b) High-level ROS encourage cell variation and conversion into malignant cells. The production of ROS and antioxidation ability in tumor cells are both increased in various degrees, but cancer cells tend to be in a higher oxidation environment. (c) Toxic-dose ROS cause cell death or apoptosis and is also the killer of cancer cells.

ROS importantly work in the physiology of the skin. As the first barrier of body, the skin protects the body against various harmful factors like pathogens, physical factors, and chemical drugs. At a low level, ROS are beneficial to maintain normal metabolism and cell growth through mediating a variety of signal transduction pathways as a second messenger [16]. They are quite essential for skin physiological processes such as cutaneous cell proliferation, dermal angiogenesis, wound healing, and skin repair [17]. Nevertheless, high-level ROS produced by various external factors (e.g., chemical toxicants, UV, IR, and pathogen infection) or internal factors (e.g., ischemia/reperfusion, inflammation, and hypoxia) would disrupt redox homeostasis in the skin, further trigger severe oxidative stress, and then cause cell membrane lipid peroxidation, eventually resulting in DNA/cell damage or variation and even carcinogenesis [18]. These may encourage cutaneous lesion appearance and tumor growth in the skin, such as melanoma, BCC, and SCC [19]. It is confirmed that ROS participate in carcinogenesis in various ways like modulating related signaling pathways, changing cell metabolism, and causing the instability of the genome and epigenome [20, 21]. However, the role of ROS in skin cancer has not been completely clarified. In addition, it is reported that a super high dose of ROS could fight cancer basing on ROS inducing cell death/apoptosis, which indicates that ROS would be a potential target of anticancer therapy. The effects of different concentrations of ROS on cells are summarized in Figure 1. Herein, we review the recent data about ROS and skin cancer to elucidate the role of ROS in carcinogenesis and their correlation. Moreover, treatments based on ROS for skin cancer, including chemotherapy, phototherapy, radiotherapy, and dietary antioxidants, are also discussed.

## 2. Role of ROS in Carcinogenesis

There are two ways for ROS to work in carcinogenesis: genotoxicity and nongenotoxicity. The former is chiefly about genotoxic substance-induced direct DNA damage, which may cause protooncogene activation, tumor suppressor gene inactivation, genomic instability, and epigenetic modifications, further leading to mutations. The latter has an indirect effect on DNA through the activation of related signaling pathways. The following are the details that ROS mediate in cancer, skin cancer in particular, through these two ways.

### 2.1. ROS-Mediated Genotoxicity in Carcinogenesis

#### 2.1.1. Genomic Instability

As one of the most potent DNA-damaging agents, ROS induce genomic instability in numerous ways. ROS, derived from mitochondrial respiratory chain complex III, greatly encourage DNA oxidative damage, not only destroying DNA bases to generate 7,8-dihydro-8-oxo-2′-deoxyguanosine (8-oxodG) but also producing spontaneous DNA double-strand breaks (DSBs), ultimately resulting in chromosomal aberrations and the accumulation of tyrosine kinase inhibitor-resistant BCR-ABL1 mutants [22]. Weyemi et al. in their reports showed that ROS-produced NOX4 played a critical role in oncogenic Ras-induced DNA damage. H-Ras continuously stimulated the overexpression of NOX4 and its functional partner p22^phox^, and thereby produced a large amount of H_2_O_2_ which would induce DNA damage and initiate carcinogenesis [23]. By activating the expression of Ras and c-Myc oncogenes, ROS promote cancer progression and invasion; Ras in turn induces ROS overproduction [24]. Recent studies revealed that NOX-derived ROS were largely responsible for the development of melanoma; NOX1/NOX4-induced ROS could trigger the invasion of melanoma through enhancing Rac1 expression, participating in the epithelial-mesenchymal transition (EMT) process, and activating the downstream signals of the AKT pathway [25]. Aydin et al. meanwhile reported that NOX2-derived ROS encouraged metastasis of melanoma cells via diminishing the effects of NK cells and lymphocytes [26]. Similarly, NOX5-derived ROS elevated the proliferation of human UACC-257 melanoma cells via stimulating HIF-1*α* expression, further enhancing new blood vessel formation and accelerating the growth and invasion of tumors [27]. Moreover, endogenous estrogen metabolite-produced ROS could cause oxidative damage and DSB production, which induce antioncogene BRCA1 mutations and prevent DNA damage repair, eventually encouraging genomic instability and tumorigenesis [28]. Normally, the tumor suppressor gene p53 plays crucial roles in DNA damage repair, cell growth/apoptosis, and tumorigenesis inhibition; however, ROS-induced mutations in p53 may spoil these functions and promote carcinogenesis including skin cancer, lung cancer, gastric cancer, and colon cancer [29–31].

#### 2.1.2. Epigenetic Changes

On the other hand, ROS-induced epigenetic instability/modification also plays an important part in carcinogenesis via the genotoxicity way. The ROS-induced epigenetic modification often manifests as a global hypomethylation of the genome and an abnormal hypermethylation in the CpG island region of some genes. ROS could promote DNA methylation to result in the silence of the tumor suppressor gene and the activation of oncogene by upregulating the expression of DNA methyltransferases (DNMTs) or by forming a new DNMT-containing complex [32]. For example, H_2_O_2_ powerfully induced the hypermethylation of CDX1 or runt domain transcription factor 3 (RUNX3) promoter and silenced these genes in colorectal cancer, which indicated that ROS could promote cancer cell proliferation by inducing tumor inhibitor gene silence [33, 34]. As the main scavenger of ROS, glutathione peroxidase 3 (GPX3) is considered to be a potent tumor suppressor in many cancers; nevertheless, GPX3 promoter hypermethylation could stop its antioxidant function in clear cell renal cell carcinoma (ccRCC), which indicates that the failure of the antioxidant system in ccRCC cells may be related to renal carcinogenesis [35]. Furthermore, ROS could promote carcinogenesis through mediating histone modifications or interfering microRNA (miRNA) dysregulation. Gene activation or inhibition caused by ROS-mediated histone modification depends on the modified amino acid residues, and histone acetylation modification is mainly coordinated by histone acetyltransferase (HAT) and histone deacetylase (HDAC), while the level of histone acetylation is always low in cancer cells; especially, the hypomethylation of histone H3K9 leads to melanoma epigenetic instability [36]. Besides, ROS-induced miRNA (such as miR-125b) dysregulation is closely implicated in skin carcinogenesis via interfering with the normal activities of key genes [37].

### 2.2. ROS-Mediated Nongenotoxicity in Carcinogenesis: Abnormal Activation of Cellular Signaling Pathways

**Figure 2:**
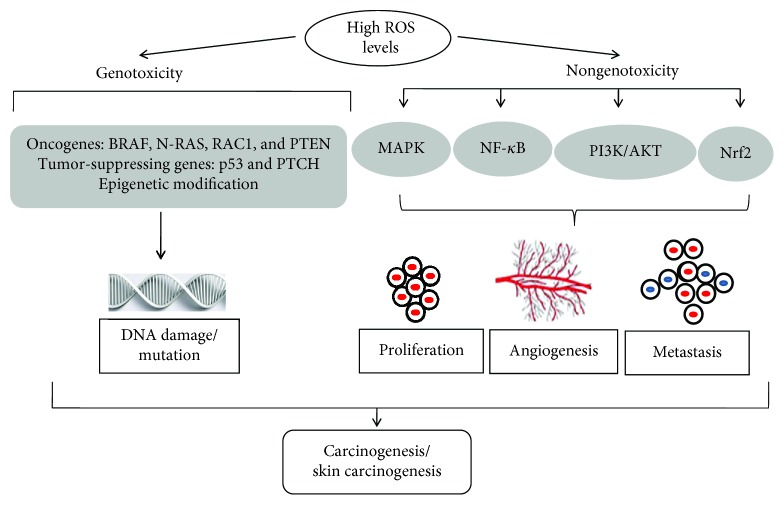
ROS crucially mediates in carcinogenesis/skin carcinogenesis. High/increased levels of ROS benefit carcinogenesis, especially the development and progression of skin cancer including melanoma, SCC, and BCC. On the one hand, they activate protooncogenes (BRAF, N-Ras, RAC1, PTEN, etc.), inactivate tumor suppressor genes (p53, PTCH, etc.), and cause epigenetic modification. These changes lead to DNA damage and mutation resulting in skin carcinogenesis in a genotoxic way. On the other hand, they trigger cancer in a nongenotoxic way, namely, through the activation of related signaling pathways, such as MAPK, NF-*κ*B, PI3K/AKT/mTOR, and Nrf2. The activation of these signaling pathways leads to the proliferation, angiogenesis, and metastasis of skin cancer cells. Together, these processes cause the occurrence of carcinogenesis/skin carcinogenesis.

Moderate-dose ROS like O_2_^•-^ and H_2_O_2_ facilitate the abnormal proliferation, metastasis, and infiltration of various tumor cells through activating multiple pathways including oxidative stress-related pathways and antioxidant stress pathways, such as the mitogen activated-protein kinase (MAPK) pathway, the phosphoinositide-3-kinase (PI3K)/protein kinase B (PKB or AKT)/mammalian target of rapamycin (mTOR) pathway, the nuclear factor-*κ*B (NF-*κ*B) pathway, and the nuclear factor erythroid 2-related factor 2 (Nrf2) pathway [38]. First, the MAPK signal pathway, consisting of the extracellular-regulated kinase (ERK), the c-Jun N-terminal kinase (JNK), and the p38 kinase isoenzyme, effectively works in mitosis, metabolism, cell proliferation, and growth, as well as apoptosis [39]. In many studies, it has been observed that elevated ROS could activate the MAPK/ERK signaling pathway and enhance the proliferation, invasion, and metastasis of tumor cells [40, 41]; most melanoma patients carried BRAF gene mutations, which might activate the MAPK/ERK signaling pathway, further promoting tumor cell proliferation through regulating the downstream signals, and ultimately leading to tumorigenesis and even tumor progression [42]. Second, the PI3K/AKT/mTOR pathway, as a classic signaling pathway, widely exists in cells to promote cell survival, inhibit apoptosis, and prevent autophagy; this pathway is overactivated in various tumor tissues and facilitates carcinogenesis and angiogenesis [43, 44]. ROS are able to activate the PI3K/AKT/mTOR pathway and mediate the proliferation and migration of tumor cells [45]. Indeed, ROS enhance the proliferation of melanoma cells via stimulating the PI3K/AKT pathway that interacts with the MAPK pathway [46]. Third, the NF-*κ*B signaling pathway is greatly activated by increased ROS in cancer cells and has a large influence on carcinogenesis [47]. Accumulating findings indicate that NF-*κ*B target genes remarkably benefit cellular survival. It has been shown that ROS could activate the NF-*κ*B signal pathway to promote the angiogenesis and progression of melanoma [48, 49]. On the contrary, the metastatic activity of malignant cells would significantly decrease when ROS-mediated NF-*κ*B activation was suppressed [50]. Fourth, Nrf2 has a dual effect of antitumorigenesis and protumorigenesis in different stages [51]. In the early stage of UV-induced skin carcinogenesis, Nrf2 activation promotes the proliferation of normal cells which greatly outnumbers precancerous cells, and prevents precancerous cell expansion and mutant transformation. Inversely in the late stage, Nrf2 activation is quite beneficial to precancerous/cancerous cell survival, due to oncogene mutations providing higher proliferation and viability for these cells via upregulating Nrf2 expression [52]. On one hand, Nrf2 facilitates carcinogenesis and cancer cell growth/proliferation; numerous studies have demonstrated that Nrf2 highly expresses in a variety of cancer cells and promotes ROS detoxification and tumorigenesis [53–55]. Meanwhile, ROS-related Nrf2 activation of macrophages increased vascular endothelial growth factor (VEGF) expression and facilitated cancer cell EMT [56]. Another study showed that the activated Nrf2 positively worked in skin tumor by protecting the protumorigenic activity of keratinocytes from ROS-induced damage and apoptosis [57]. On the other hand, Nrf2 has an antitumorigenesis effect. The decreased Nrf2 spoils the impaired antioxidant defense system, which may increase the incidence of skin cancer including melanoma, SCC, and BCC [58]. Similarly, Nrf2 knockout mice were more susceptible to SCC than controls [59]. More importantly, it has been demonstrated that Nrf2 knockout mice could be subjected to persistent DNA damage, substantial extracellular matrix degradation, and serious inflammation; inversely, the activation of Nrf2 benefited the prevention of skin carcinogenesis in Nrf2 knockout mice [60]. Therefore, the activation of Nrf2 would be a promising strategy for the treatment and prevention of skin carcinogenesis by improving antioxidant capacity to protect cells from oxidative damage. Many Nrf2-activating compounds are beneficial to the prevention of skin cancer, and they contain curcumin, quercetin, and resveratrol [61]. Besides, other redox signaling pathways are implicated in carcinogenesis and tumor development, containing Wnt/*β*-catenin, TGF-*β*/Smad, etc. [62, 63].  Figure 2 sketches the role of ROS in carcinogenesis, especially in skin carcinogenesis.

## 3. Relationship between ROS and Skin Cancer

ROS could promote cutaneous carcinogenesis and cancer progression by mediating related pathways. But until now, the mechanism of ROS influencing skin cancer has not been completely clarified and only part of them have been explored. Herein, we endeavour to elucidate the relationship between ROS and skin cancer basing on the related data.

Melanoma, derived from melanocytes, is a highly invasive tumor with the incidence increasing yearly [64]. Excessive UV exposure is a crucial susceptibility factor, and UV-produced substantial ROS contribute to nuclear DNA damage. ROS not only trigger the occurrence and development of melanoma by way of genotoxicity and some specific signaling pathway activation but they also cause oncogene activation or tumor-suppressing gene inactivation in melanoma consisting of BRAF, c-Myc, p53, and Ras genes. N-Ras is upstream of the MAPK pathway, and its mutation commonly occurs in melanoma, which contributes to cancer cell proliferation [65]. Moreover, ROS also drive the stable expression of HIF-1*α* to activate the Met protooncogene, which facilitates the proliferation of the extracellular matrix, angiogenesis, and the proliferation and metastasis of melanoma cells [66]. Other oncogenes, RAC1 in particular, are associated with an increased risk of melanoma [67]. The activation of RAC1 depends on the levels of ROS and determines the ability of the migration and invasion of B16 melanoma cells which could be weakened by the suppression of ROS-mediated Rac-1 activation [68]. Apart from the abovementioned factors, other signaling pathways especially the PI3K/AKT pathway and NF-*κ*B are implicated in the initiation and progression of melanoma [69]. Therefore, ROS are crucially responsible for the occurrence and development of melanoma through inducing related gene mutations and activating a serial of signaling pathways [70]. However, too much ROS generation would encourage apoptosis, which may become a useful vehicle to kill melanoma cells. Subsequently, these will be discussed in the follow-up part of treatments.

Originating from the basal cells near the epidermis-dermis junction, BCC primarily occurs in middle-aged and elderly people, and its lesions mostly appear in exposed areas such as the head, face, and neck. Many factors (e.g., UV, some harmful chemicals, and IR) may trigger BCC initiation, among which UV exposure is a particularly important one [71]. UV-induced ROS could promote the occurrence and development of BCC by generating 8-oxoG and reducing hOGG1 [6]. The imbalance of ROS would encourage skin inflammation, abnormal metabolism, and decreased immunity, which eventually leads to cell mutation and carcinogenesis. Compared with control individuals, there was a high level of MDA in BCC patients, with a reduction of antioxidant components, which enhanced the occurrence of BCC [72]. In the same way, the expression of oxidative DNA damage product 8-oxoG increased, while the levels of antioxidation defenses (e.g., hOGG1, CAT, GPx, and Nrf2) decreased in BCC tissues [73]. UV radiation and oxidative stress facilitate the membrane receptor PTCH gene mutations, which would result in abnormal activation of the hedgehog signaling pathway; in turn, PTCH gene activation and the abnormal activation of the hedgehog signaling pathway are closely involved in the pathogenesis of BCC [74].

As an extremely common type of skin cancer, SCC is derived from keratinocytes and attacks the upper layer of the skin. Excessive UV exposure is a main causative factor for SCC, and UV-induced ROS play a crucial role in carcinogenesis and in the promotion of SCC, while ROS-mediated oxidative stress exacerbates the oxidative damage of DNA, protein, and lipid, further magnifying the progression and invasion of SCC [75, 76]. UV-produced ROS in skin always act as an essential role in inducing p53 mutation. As a tumor suppressor protein, p53 conserves genome stability, maintains normal cell growth, and prevents cell malignant transformation. Once DNA is damaged, p53 would accelerate DNA replication and repair by activating DNA repair proteins, prevent cell growth from arresting the cell cycle, and initiate programmed cell death if DNA damage is irreparable [77]. In humans, *TP53* is the major gene encoding p53, and its mutational inactivation most frequently occurs in skin cancers, e.g., SCC and BCC, especially in SCC [78]. Liu et al. discovered that in the absence of p53 function, inhibition of p38*α* MAPK activity enhanced A431 SCC cell proliferation and drove UV-induced skin carcinogenesis in p53^−/−^/SKH-1 mice, which was closely associated with increased ROS/NOX2 as well as aberrant p53 [79]. In addition, accumulative ROS could induce *PTEN* gene mutation and inactivation in oxidative damage-related skin cancers, SCC in particular. *PTEN*, a tumor suppressor gene, negatively regulates the PI3K/AKT pathway and often undergoes mutations, deletions, or silencing in many cancers [80]. Ming et al. showed that *PTEN* expression markedly decreased in SCC, suggesting a critical effect of *PTEN* in skin carcinogenesis and skin cancer procession [81].

## 4. Treatments for Skin Cancer Targeting ROS

**Figure 3:**
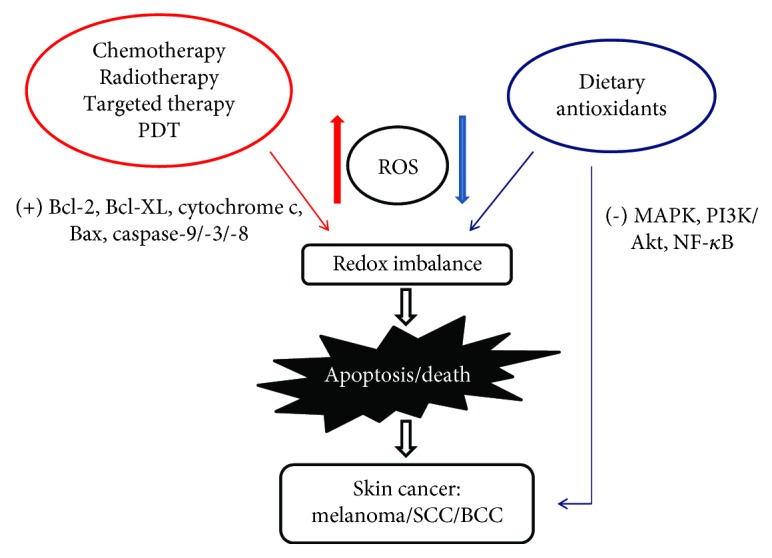
Therapies for skin cancer basing on ROS. There are many treatments for skin cancer in a ROS-targeted way, including chemotherapy, radiotherapy, targeted therapy, and PDT. These therapies cause toxic-dose ROS production and then lead to redox imbalance, further activating Bcl-2, Bax, and caspase-9 as well as other pathways to induce skin cancer cell death/apoptosis. On the other hand, dietary antioxidants reduce the production of ROS by inhibiting signal pathways such as MAPK, PI3K-Akt, and NF-*κ*B to prevent and control skin cancer (melanoma, SCC, and BCC). In fact, the mechanism of the ROS-based treatment of skin cancer is often interactive. (+) indicates activation and (-) indicates inactivation.

There are many therapies for skin cancer, including surgery, chemotherapy, radiotherapy, photodynamic therapy (PDT), and molecular targeting therapy, etc., among which surgery is the most common and important one [82]. Nevertheless, numerous studies have shown that higher-level ROS and redox imbalance often emerge from cancer cells, which could cause multidrug resistance (MDR) and immunosuppression of cancer cells and thereby make it quite difficult to control tumors [83]. At the same time, when the skin cancer occurs at a special site, or the lesions are too large or many to operate, or the patient is too old to tolerate surgery, or distant metastasis of tumors occurs, other medical approaches such as radiotherapy, PDT, and/or chemotherapy may be better alternatives [84]. Given that ROS play an important role in promoting skin cancer, many ROS-targeted treatments would be well developed (shown in Figure 3).

### 4.1. Medical Treatments for Skin Cancer Basing on ROS

Skin cancer cells have a higher oxidative environment, and ROS have a double effect on cutaneous carcinoma. On the one hand, reducing ROS production contributes to inhibiting skin cancer; but on the other hand, diminishing antioxidant enzymes may enhance toxic-dose ROS production and weaken the body's antioxidant defense, eventually inducing cancer cell death. Thus, more and more ROS-targeted therapies/drugs have been discovered in recent years.

Related researches have shown that celecoxib combined with 5-fluorouracil (5-FU) could suppress the phosphorylation of AKT to reduce the proliferation of SCC cells via producing a large amount of ROS in a dose-dependent manner. Once FU is converted into FU deoxynucleotides in cells, it would block thymidine nucleotide synthetase and inhibit DNA synthesis. FU meanwhile interferes with the synthesis of RNA to resist tumors. Moreover, increased ROS cause oxidative damage and then result in the breakage of NMSC cell membrane lipids, proteins, and DNA strand chain [85, 86]. The targeted inhibitors dabrafenib and trametinib were used to treat melanoma and SCC mainly by involving ROS overproduction and caspase-activated apoptosis [87]. Daniel et al. also found that the combined therapy of vemurafenib and potassium channel inhibitor TRAM-34 decreased ERK phosphorylation and significantly increased intracellular ROS levels, which stimulated caspase-3 and other proapoptotic pathways and decreased the mitochondrial membrane potential, further leading to the apoptosis of melanoma cells [88]. For example, targeting BRAF gene drugs vemurafenib and dabrafenib could inhibit the growth and division of BRAF-mutated metastatic melanoma cells via blocking the MAPK signaling pathway and upregulating ROS [89]. Meanwhile, the MEK inhibitor trametinib combining with dabrafenib significantly enhanced the therapeutic effect on melanoma in the presence of high-level ROS [90]. Besides, chaetocin derived from the *Chaetomium* species has a powerful antitumor proliferative activity. It significantly inhibited melanoma cell proliferation and promoted its apoptosis via increasing cellular ROS, decreasing the mitochondrial membrane potential and activating the caspase-9/3 pathway [91]. Nevertheless, Yu et al. and Wang et al. discovered that the ROS-responsive gel scaffold that they created in their study could break immune tolerance and enhance immune response to melanoma through reducing the level of local ROS and inhibiting the programmed death-ligand 1 (PD-L1) [92, 93].

PDT is a phototherapy based on the accumulation of photosensitizers in the body and the irradiation of light with a specific wave length, which can generate substantial ROS to produce cytotoxicity and kill cancer cells. Currently, 5-methylaminolevulinic acid (MAL) and 5-aminolevulinic acid (ALA) are both extremely common photosensitizers in PDT, and PDT has been widely used to treat skin tumors, e.g., SCC, BCC, and Bowen's disease. The presence of either MAL or ALA in the body may be converted into protoporphyrin IX (PpIX) with strong photosensitivity, which produces substantial ROS to kill cancer cells after irradiation with adequate-wavelength light, while neighbouring normal cells are scarcely affected [94]. However, PDT has a large limitation in skin cancer due to the infiltration of photosensitizers into deep skin tissue. To overcome this deficiency, some improvements, including pretreatment with a laser or a microneedle and encapsulating the photosensitizer in nanoparticles and combining with drugs, are made to enhance PDT efficacy in skin cancer [95]. Others, like indoline-fused-triazole-mediated PDT can increase ROS production and enhance apoptosis-related protein expression, thereby inducing BBC cell death [96].

Furthermore, there are other ways for skin cancer treatment targeting ROS. Typically, radiotherapy is an effective vehicle in the management of skin cancer in recent decades [97]. Via locally producing and releasing a large quantity of ROS, radiotherapy can cause violent oxidative eruptions to kill tumor cells and make solid tumor smaller [98]. Recently, it has been demonstrated that some ROS-inducers are conducive to enhancing the sensitivity of skin cancer cells to IR through a ROS-mediated manner. Selenadiazole derivatives, for example, could increase the sensitivity of A375 human melanoma cells to X-ray by the induction of ROS-mediated DNA damage and AKT inactivation. Besides, IR benefits more ROS generation, G2/M phase arrest, and melanoma cell apoptosis [99].

### 4.2. Dietary Antioxidants for Skin Cancer Basing on ROS

Dietary antioxidants are widely distributed in fruits, vegetables, grain, herbs, spices, and other foods, which are rich in vitamins, minerals, polyphenols, and flavonoids. Dietary antioxidants possess various antineoplastic activities: antiproliferation, anti-inflammation, immune regulation, antiangiogenesis, and inhibition of metastasis [100, 101]. Dietary intake of vitamins, including vitamin C, vitamin E, selenium, and vitamin A, is inversely proportional to the risk of cancer and prevents skin carcinogenesis as antioxidant micronutrients [102]. When UV-induced ROS are beyond the antioxidant defense, oxidative stress occurs; nevertheless, these vitamins could effectively eliminate ROS and prevent oxidative stress through strengthening the antioxidant defense, ultimately protecting the skin against UV-induced cancer [103]. Moreover, polyphenols are a group of natural substances with excellent biological properties and have become potent dietary-preventive agents against cancer. The polypodium leucotomos extract (PL), a strong antioxidant with a high-content phenolic compound, is able to prevent and control skin cancer mainly by inhibiting UV-induced ROS production, suppressing NF-*κ*B activation, and activating the p53 protein [104]. Heo et al. found that the decrease of Nrf2 expression and the antioxidant defense ability in resveratrol-treated melanoma cells encouraged the generation of a large amount of ROS and endoplasmic reticulum stress, then triggered the occurrence of oxidative stress; in turn, the increased ROS and oxidative stress further inhibited the growth and proliferation of melanoma cells by downregulating the Bcl-2 protein level and upregulating the Bcl-2-related X protein expression [105]. As a member of the flavonoid family, quercetin is excellent in strengthening the antioxidant defense via removing H_2_O_2_, O_2_^•-^, and ^•^OH and has a powerful anticancer effect on skin cancer through regulating molecular mechanisms, e.g., inhibiting activation of the MAPK, PI3K-Akt/PKB, and NF-*κ*B signal pathways [106]. Another natural flavonoid, caffeic acid n-butyl ester (CAE), stimulates the accumulation of toxic ROS and the decrease of MMP in A431 skin cancer cells to inhibit the PI3K/AKT/mTOR signaling pathway and thus induce cancer cell apoptosis [107]. Lee et al. meanwhile showed that the flavonoid Cudraflavone C was a novel natural drug for the treatment of melanoma; this drug could activate the phosphorylation of MAPKs (p38, ERK, and JNK) and increase the expression of apoptosis proteins (Bax, cytochrome c, caspase-9, and caspase-3/7) to induce the apoptosis of melanoma cells by increasing mitochondrial ROS production [108]. Proanthocyanidins, a group of flavonoids derived from grapes, apples, bilberry, cranberry, and other plants, have potent abilities of deducing the proliferation and invasion of tumor cells through the production of toxic-dose ROS and inhibition of MMP-2/9 expression, eventually preventing skin carcinogenesis, especially SCC [109]. Other studies also have demonstrated that proanthocyanidins, owing to their strong antineoplastic and antiangiogenic properties in cancers, could downregulate VEGF expression, suppress endothelial cell migration, and lessen vascularization via attenuating the phosphorylation of Akt, ERK, and p38 MAPK [110]. In addition, dietary antioxidants like some Thai plants have protective effects against UV-induced skin cancer [111]. Overall, dietary antioxidants have diverse beneficial properties and provide a protection against skin cancer through regulating some molecular mechanisms between ROS and cancer.

Figure 3 summarizes these ROS-targeted treatments on skin cancer.

## 5. Conclusion and Future Perspective

Taken together, there is convincing evidence to support the critical role of ROS in cutaneous carcinogenesis and skin cancer progression. Increased ROS contribute to DNA damage and epigenetic instability, metabolic adaptation, cancer cell proliferation and migration, and cell death in some cases. In recent years, it has become a research hot spot in the tumor therapy field whether to focus on antioxidation or promote oxidation. In this review, a series of mechanisms in ROS-mediated skin cancers have been discussed, including protooncogene activation, tumor suppressor gene inactivation, genomic instability/mutations, and epigenetic modifications, as well as multiple related signaling pathways; several therapeutic approaches targeting ROS, like PDT, radiotherapy, and dietary therapy, are also introduced. Although the relationship between ROS and skin carcinogenesis has been largely elucidated, how they specifically regulate each other needs further research. We look forward to finding the balance between ROS and skin carcinogenesis in the near future and searching a reliable and effective method for the treatment of skin cancer.
